# The oncogenic properties of EWS/WT1 of desmoplastic small round cell tumors are unmasked by loss of p53 in murine embryonic fibroblasts

**DOI:** 10.1186/1471-2407-13-585

**Published:** 2013-12-09

**Authors:** Pratiti Bandopadhayay, Anissa M Jabbour, Christopher Riffkin, Marika Salmanidis, Lavinia Gordon, Dean Popovski, Lin Rigby, David M Ashley, David N Watkins, David M Thomas, Elizabeth Algar, Paul G Ekert

**Affiliations:** 1 Department of Pediatric Hematology/Oncology, Dana-Farber Cancer Institute and Boston Children’s Hospital, Boston, MA, USA; 2 University of Melbourne, Royal Parade, Parkville, Melbourne, Victoria 3052, Australia; 3 Walter & Eliza Hall Institute, Royal Parade, Parkville, Victoria 3052, Australia; 4 Murdoch Childrens Research Institute, Royal Children’s Hospital, Flemington Rd, Parkville, Victoria 3052, Australia; 5 Department of Medicine, Deakin University, Geelong, Victoria 3220, Australia; 6 Monash Institute of Medical Research, Monash University, Clayton 3168, Victoria, Australia; 7 Sir Peter MacCallum Department of Oncology, University of Melbourne and Peter MacCallum Cancer Centre St Andrew’s Place, East Melbourne 3002 Victoria, Australia

## Abstract

**Background:**

Desmoplastic small round cell tumor (DSRCT) is characterized by the presence of a fusion protein EWS/WT1, arising from the t (11;22) (p13;q12) translocation. Here we examine the oncogenic properties of two splice variants of EWS/WT1, EWS/WT1-KTS and EWS/WT1 + KTS.

**Methods:**

We over-expressed both EWS/WT1 variants in murine embryonic fibroblasts (MEFs) of wild-type, p53^+/-^ and p53^-/-^ backgrounds and measured effects on cell-proliferation, anchorage-independent growth, clonogenicity after serum withdrawal, and sensitivity to cytotoxic drugs and gamma irradiation in comparison to control cells. We examined gene expression profiles in cells expressing EWS/WT1. Finally we validated our key findings in a small series of DSRCT.

**Results:**

Neither isoform of EWS/WT1 was sufficient to transform wild-type MEFs however the oncogenic potential of both was unmasked by p53 loss. Expression of EWS/WT1 in MEFs lacking at least one allele of p53 enhanced cell-proliferation, clonogenic survival and anchorage-independent growth. EWS/WT1 expression in wild-type MEFs conferred resistance to cell-cycle arrest after irradiation and daunorubicin induced apoptosis. We show DSRCT commonly have nuclear localization of p53, and copy-number amplification of *MDM2*/*MDMX*. Expression of either isoform of EWS/WT1 induced characteristic mRNA expression profiles. Gene-set enrichment analysis demonstrated enrichment of WNT pathway signatures in MEFs expressing EWS/WT1 + KTS. Wnt-activation was validated in cell lines with over-expression of EWS/WT1 and in DSRCT.

**Conclusion:**

In conclusion, we show both isoforms of EWS/WT1 have oncogenic potential in MEFs with loss of p53. In addition we provide the first link between EWS/WT1 and Wnt-pathway signaling. These data provide novel insights into the function of the EWS/WT1 fusion protein which characterize DSRCT.

## Background

Desmoplastic small round cell tumor (DSRCT) is a highly aggressive tumor that most commonly affects adolescents and young adults [1]. DSRCT has a dismal prognosis, and novel therapeutic approaches are desperately needed. The EWS/WT1 translocation t(11;22)(p13;q12) is pathognomonic for DSRCT [2], and most commonly fuses exon 7 of *EWS* to exon 8 of *WT1* although break-points may vary [3,4]. DSRCT are classified as soft tissue sarcomas and have evidence of co-expression of epithelial markers (cytokeratin), mesenchymal markers (desmin and vimentin) and neuronal markers (neuron-specific enolase), with the cell of origin yet to be determined [1].

The EWS/WT1 protein comprises the N-terminal domain of EWS1 fused to zinc finger 2 of the WTI protein [2]. WT1 contains a regulatory domain and four zinc fingers required for DNA binding and RNA modulation functions. Alternate splicing in exon 9 of WT1 and EWS/WT1 generates an insertion of three amino acids lysine, threonine and serine (KTS) between zinc fingers 3 and 4, producing + KTS and –KTS isoforms [5]. While both EWS/WT1-KTS and EWS/WT1 + KTS have been described in DSRCT, it remains unclear whether the oncogenic properties of EWS/WT1 derive from one or other isoform and existing data is contradictory [5,6]. Although EWS/WT1-KTS has been reported to transform NIH3T3 cells [5], EWS/WT1 + KTS has not been shown to have oncogenic properties.

Most published data on the t(11;22)(p13;q12) translocation have focused on EWS/WT1-KTS. Reported transcriptional targets regulated by EWS/WT1 include PDGFA [6], IGFR1 [7], TALLA-1 [8] and BAIAP3 for EWS/WT1-KTS [9] and LRRC15 for EWS/WT1 + KTS [10]. Only one gene, ENT4, has been reported to be regulated by both [11]. These targets have been identified in immortalized or cancer cell lines such as NIH3T3 cells, and osteosarcoma cell lines. The lack of patient derived DSRCT cell lines and paucity of patient derived tumor samples reflect the rarity of the tumor. Lack of *in vivo* models have hampered efforts to identify potential therapeutic targets.

In this project we sought to examine the functional effects of over-expression of EWS/WT1-KTS and EWS/WT1 + KTS in primary murine embryonic fibroblasts. We show for the first time that oncogenic properties of both isoforms are unmasked by loss of p53 function. Further we provide the first links between the EWS/WT1 fusion protein and canonical Wnt-pathway activation. These data provide novel insights into the potential oncogenic roles of EWS/WT1 in DSRCT.

## Methods

Ethics approval was granted by the relevant human and/or animal ethics research committees of the Royal Children’s Hospital, Murdoch Childrens Research Institute and Walter Eliza Hall Institute of Medical Research, Victoria, Australia.

### Generating MEFs that express EWS/WT1 and confirming expression of EWS/WT1

MEFs were generated from E14.5 embryos of C57BL6 mice, and from p53-knockout mice [12]. p53 knock- out mice were a kind gift from Dr Bouillet, Melbourne. Full-length human EWS/WT1-KTS, EWS/WT1 + KTS (gift from Dr. Haber, Boston) or eGFP were cloned into the pF5xUAS-SV40-puromycin lentiviral vector [13]. Cells were infected with GEV16 lentivirus and pF5xUAS-SV40 containing EWS/WT1 or eGFP. Expression of EWS/WT1 was confirmed following selection. Transcripts were also cloned into a doxycycline-regulated Tet-Off lentiviral vector, pF 7× tOp MCS RS PGK Hygro TetR VP16 (Gift from Dr. Silke, Melbourne) [14]. Lentivirus was generated and cells infected as previously described [14]. The dose of 4-OHT was 0.1 μM and the dose of doxycycline was 500 ng/ml.

Whole cell lysates were generated using RIPA buffer with phosphatase inhibitor and protease inhibitor cocktail at a concentration of 1×10^4^ cells/μL and boiled for 10 minutes in protein sample buffer. Samples were electrophoresed on 10% or 12% SDS page gels (BioRad) and transferred to nitrocellulose for antibody detection.

Proteins were detected by chemiluminescence using an ECL kit (Amersham, UK). Antibodies used (1:1000 dilution) were anti-p21 (Santa Cruz Biotechnology, CA, USA: Cat number SC-271532), anti p53 (Leica Biosystem’s Novocastra, IL, USA Cat number: NCL-p53-CM5P), anti-p27 (Cell Signaling Cat number :2552), anti-rabbit IgG HRP (1:10000) (GE Healthcare Life Sciences, NY, USA Cat number: Amersham NA934) and anti-mouse IgG HRP (1:10000) (Sigma-aldrich, MO, USA Cat number: HA2304). Anti-WT1 (Santa Cruz C-19) was used in a 1:500 dilution.

### Cell proliferation and immortalisation assays

Equal numbers of freshly generated MEFs expressing eGFP, EWS/WT1-KTS or EWS/WT1 + KTS were plated on 15 cm gelatinized plates DMEM/10% FCS and maintained in selection. Cells were split every three to four days (1:4 to 1:5) and number of live cells counted.

### Anchorage independent clonogenic assays

1000 cells of p53^+/+^, p53^+/-^ and p53^-/-^ backgrounds expressing either eGFP (vector control), EWS/WT1-KTS or EWS/WT1 + KTS were plated in DMEM, 20% FCS and 0.3% soft agar in six-well plates and incubated for 14 days. Colonies greater than 2 mm were counted. Three independent experiments were performed.

### Serum deprivation assays

10,000 cells of p53^+/+^, p53^+/-^ and p53^-/-^ backgrounds expressing either eGFP, EWS/WT1-KTS or EWS/WT1 + KTS were plated on gelatinized 10 cm plates in DMEM with either 1% or 2% FCS for 14 days and then fixed with 1% glutaraldehyde for 30 minutes and stained with crystal violet. Colonies greater than 5 mm were counted. Three independent experiments were performed.

### Cell viability assays assessing response to daunorubicin therapy

1000 cells per well of p53^+/-^ and p53^-/-^ background expressing either eGFP, EWS/WT1-KTS or EWS/WT1 + KTS were plated in 96-well plates. Cells were treated with varying doses of daunorubicin diluted in DMEM/10%FCS. At 24 hours a formazoan dye based (WST-1) (Roche Applied Science, IN, USA Cat number:11644807001) cell-viability assay reagent [15] was added (1:10) and incubated for one hour at 37°C. Three independent experiments were performed.

### Cell-cycle assay following treatment with radiation

Wild type MEFs expressing either eGFP, EWS/WT1-KTS or EWS/WT1 + KTS were treated with 10 Gy gamma irradiation. Cells were lysed in hypo-PI buffer (0.1% Na_3_Citrate in ddH_2_O, 0.1% TritonX-100, 50 μg/ml Propidium Iodide (Sigma), 25 μg/ml RNase A) and nuclear staining of PI analysed by flow cytometry. Cell-cycle analysis was performed on ModFit LT analytical software (Verity Software House, ME, USA). Six independent pools of MEFs were tested over two experiments.

### Illumina microarray analysis

mRNA (DNase treated) from pools of four independent embryos, expressing eGFP, EWS/WT1-KTS or EWS/WT1 + KTS was extracted using the Qiagen RNeasy Mini Kit (Qiagen Sciences, MD, USA Cat number:74104). Samples were labeled and hybridized to Illumina MouseWG-6_V2 Expression BeadChips by the Australian Genome Research Facility (AGRF, Melbourne, Australia).

The unnormalised sample probe and control probe profiles were exported from GenomeStudio (v1.6.0). Analysis was carried out using the statistical programming language R (version 2.13.0) using packages from the Bioconductor project [16]. Data quality was confirmed using Bioconductor packages arrayQualityMetrics and lumi [17-19]. Normexp-by-control background correction, quantile normalization and log2 transformation was performed using the limma package [20]. Probes that failed to achieve a GenomeStudio detection p-value of 0.05 on any array were deemed to be not expressed, and removed from subsequent analyses. Probes were re-annotated using the ReMOAT annotation tables [20].

A linear model was fitted to test for differential expression between primary MEFs expressing eGFP, EWS/WT1-KTS or EWS/WT1 + KTS. Array weights were calculated to estimate relative quality weights for each array [21]. A hypergeometric test was carried out to compute p-values for over or under representation of gene ontologies [22].

For analyses of gene sets enriched among samples, gene set enrichment analysis (GSEA) [23,24] was performed with the C2 canonical pathway (CP) gene sets from MSigDB [23] with the addition of two WT1 gene sets [25] using standard parameters and gene-set permutations.

Our data have been deposited in NCBI’s Gene Expression Omnibus [26] are accessible through GEO Series accession number GSE42649 (http://www.ncbi.nlm.nih.gov/geo/query/acc.cgi?acc=GSE42649).

### P53 sequencing and copy number analysis of MDM2 and MDM4

Genomic DNA from tumours were screened for *p53* mutations using high resolution melting analysis with or without Sanger DNA sequencing as previously reported [27,28]. Quantitative PCR was used to measure *MDM2* and *MDM4* copy-number as previously described [29].

### Wnt qPCR assay

RNA was extracted from five DSRCT samples and also from one patient derived human fibroblast cell line. qPCR of WNT pathway members was performed using the RT^2^ Human WNT signaling pathway array (Qiagen). Data was normalized to house-keeping genes and fold change calculated using the ΔΔCt method.

### Immunohistochemistry

Paraffin sections of DSRCT were de-paraffinised using xylene and ethanol. Antigen retrieval was performed with 10nM sodium citrate pH 6. Antibodies used were anti-β catenin (Millipore cat 06–734) and anti-p53: (Dako cat p235189, clone 318-6-11) at a 1:100 dilution. Detection was with Vectastatin Elite ABC Kit (Vector Laboratories, CA, USA Cat number: pk-6101).

## Results

### EWS/WT1 (-KTS or + KTS) increases the rate of cell proliferation of SV40 transformed MEFs

**Figure 1:**
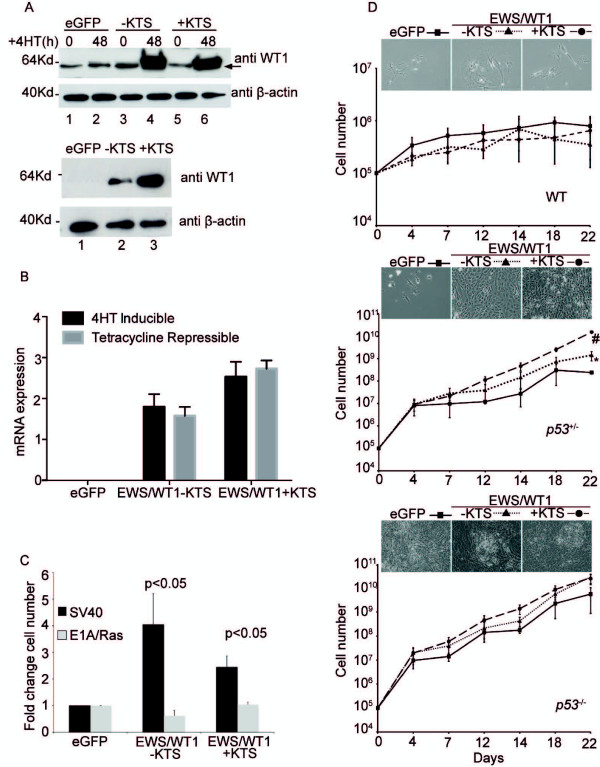
**EWS/WT1-KTS and EWS/WT1 + KTS expression co-operates with loss or inactivation of p53 to transform MEFs. (A)** Western blots of lysates from SV40 transformed MEFs expressing either eGFP, EWS/WT1-KTS or EWS/WT1 + KTS under the control of a 4-OHT inducible promoter 48 hours after 4-OHT treatment (upper panel) or a doxycycline repressible promoter without doxycycline (lower panel). The anti-WT1 antibody detects a Cterminal-epitope in WT1. Arrow indicates EWS/WT1 to distinguish it from endogenous WT1. A non-specific band of similar size to EWS/WT1 was observed in SV40-transformed, but not untransformed MEFs. **(B)** qPCR analysis of mRNA expression of eGFP, EWS/WT1-KTS or EWS/WT1 + KTS in MEFs induced by either the 4-OHT inducible or tetracycline repressible expression systems. **(C)** Fold change in cell number 14 days after MEFs transformed by either SV40 or EIA/RAS were infected with eGFP, EWS/WT1-KTS or EWS/WT1 + KTS using the 4OHT inducible system in SV40 transformed cells, and the doxycycline repressible system in EIA/RAS transformed cells. Cells were plated at equal densities and counted and re-plated at the same dilution every 3–4 days. Data represent the mean ± SEM of three independently generated pools of MEFs tested in three independent experiments. **(D)** MEFs derived from littermate wildtype (upper panel), p53+/- (middle panel) and p53-/-(lower panel) mice were infected with doxycycline repressible eGFP, EWS/WT1-KTS or EWS/WT1 + KTS and then plated at equal density. Cells were counted and re-plated on the indicated days. Values are mean ± SEM of three independently generated and infected pools of MEFs tested over three independent experiments. # denotes a p value of 0.003 and * denotes a p value of 0.004 with Student’s t-test comparing eGFP to EWS/WT1–KTS and eGFP to EWS/WT1 + KTS. Representative images of morphology of MEFs at 22 days are shown.

We used two lentiviral expression systems to over-express EWS/WT1–KTS and EWS/WT1 + KTS in primary murine fibroblasts (MEFs). These systems permitted EWS/WT1 expression to be induced by the addition of 4-Hydroxy Tamoxifen (4-OHT) to cell cultures or for repression of EWS/WT1 expression by the addition of doxycycline. Expression of both + KTS and –KTS isoforms was confirmed in both lentiviral systems using Western blotting (Figure 1A) and qPCR for mRNA expression (Figure 1B). eGFP was included as a control.

Having established reliable, inducible EWS/WT1 expression, we examined the effect of EWS/WT1 expression on proliferation of MEFs transformed either with SV40 large T antigen or E1A/RAS. Rate of cell proliferation was measured by cell counts over a 14-day time course and fold-change determined (Figure 1C). Expression of either EWS/WT1 isoform increased proliferation rates compared to eGFP controls in multiple, independent pools of SV40 large T antigen transformed MEFs (p value <0.05) but not E1A/RAS transformed MEFs. The magnitude of the increase was modest because these cells are already transformed, but robustly repeatable. In all cell lines tested and in all independent experiments, EWS/WT1 functioned to promote proliferation in SV40 large T antigen transformed but not E1A/RAS transformed MEFs. These data indicate that both isoforms of EWS/WT1 increase cell proliferation specifically in cells transformed with SV40 large T antigen. This suggests EWS/WT1 may co-operate with the loss of specific tumor suppressor pathways.

### EWS/WT1 oncogenic functions are evident in cells lacking one or both alleles of p53

The increased cell proliferation observed in MEFs transformed by SV40 expressing EWS/WT1 led us to hypothesize that EWS/WT1 co-operates with loss of p53 to promote proliferation. To investigate this, we infected freshly isolated MEFs from E14.5 embryos derived from crosses of p53^+/-^ mice, with lentivirus encoding either EWS/WT1 isoform or eGFP. For these, and subsequent experiments the doxycycline repressible lentiviral expression system was used, due to increased infection efficiency compared to the 4-OHT-inducible system.

Cells were continuously cultured, counted and re-plated every third day over a three-week time course (Figure 1D). In wild-type MEFs, neither EWS/WT1 isoform was sufficient to permit unrestricted division or the development of foci (Figure 1D upper panel), and wild type cells were unable to be maintained in culture beyond three weeks. In contrast, in p53^+/-^ MEFs both EWS/WT1 + KTS and EWS/WT1–KTS induced the development of foci and significantly increased the rate of proliferation compared to eGFP controls (Figure 1D middle panel). eGFP expressing p53^+/-^ cells eventually stopped proliferating while those expressing EWS/WT1 did not. In p53^-/-^ cells, whilst foci formed independently of infection with EWS/WT1, they were more frequent and the proliferation rate was increased in cells expressing either EWS/WT1 isoform (Figure 1D lower panel). p53^+/-^ cells expressing EWS/WT1-KTS or EWS/WT1 + KTS could be maintained in culture indefinitely while those expressing eGFP could not. These data confirm the ability of EWS/WT1 to induce foci formation and increase proliferation rates of MEFs is revealed by the loss of at least one copy of *p53*.

**Figure 2:**
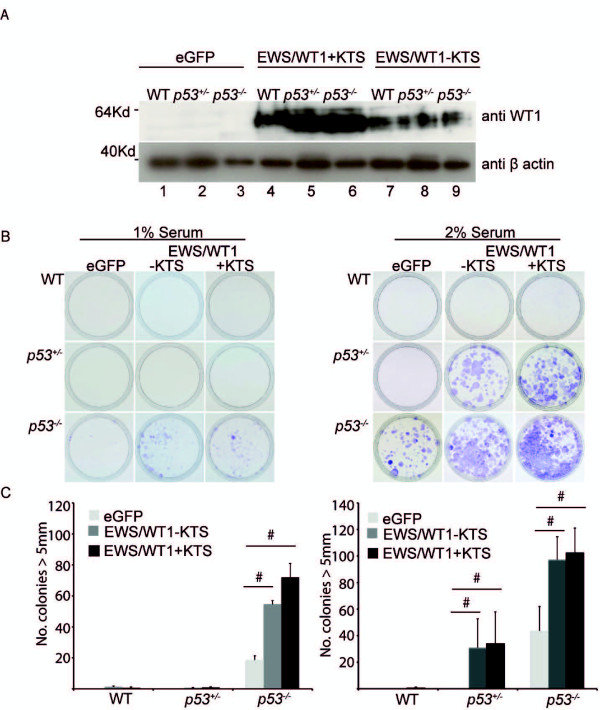
**EWS/WT1-KTS and EWS/WT1 + KTS enhances clonogenicity in p53**^**+/- **^**or p53**^**-/- **^**MEFs in reduced serum. (A)** Western blot analysis of lysates derived from wild-type, p53^+/-^, and p53^-/-^ background MEFs which were infected with doxycyline repressible eGFP, EWS/WT1-KTS or EWS/WT1 + KTS lentiviral particles, showing expression of EWS/WT1 in the absence of doxycycline. **(B)** Representative images from one experiment showing MEF colonies of the indicated genotype expressing eGFP, EWS/WT1-KTS or EWS/WT1 + KTS, plated at 1× 10^5^ cells per plate and cultured in 1% or 2% fetal calf serum. After 14 days cells were fixed with glutaraldehyde and stained with crystal violet. **(C)** Mean number of colonies (from three independent experiments) greater than 5 mm of cells cultured in 1% (left panel) or 2% (right panel) serum on day 14. Error bars show ± SEM from three independent experiments. # denotes a p value of <0.05 as determined by Student’s t-test comparing eGFP to EWS/WT1-KTS and eGFP to EWS/WT1 + KTS.

We next determined whether EWS/WT1 could promote colony formation after serum deprivation. Equal numbers of wild-type, p53^+/-^ and p53^-/-^ MEFs expressing EWS/WT1 + KTS, EWS/WT1-KTS or eGFP were cultured in media containing 1% or 2% fetal calf serum (Figure 2). Expression of EWS/WT1-KTS and EWS/WT1 + KTS was confirmed by western immunoblotting (Figure 2A). After 14 days, cells were fixed and stained with crystal violet (Figure 2B) and number of colonies greater than 5 mm diameter counted (Figure 2C). In 1% serum, EWS/WT1 over-expression significantly increased colony numbers in p53^-/-^ MEFs compared to eGFP controls, but no effect was observed in p53^+/-^ or wild-type MEFs. In 2% serum, EWS/WT1 expressing cells also rescued p53^+/-^ MEFs, which were able to form colonies. Under these less stringent conditions, EWS/WT1 + KTS and EWS/WT1–KTS maintained the viability of a proportion of p53^+/-^ cells and permitted proliferation. These data indicate that both EWS/WT1 isoforms can protect cells from serum deprivation and enhance colony formation in limiting serum concentrations in MEFs lacking one or both copies of p53.

**Figure 3:**
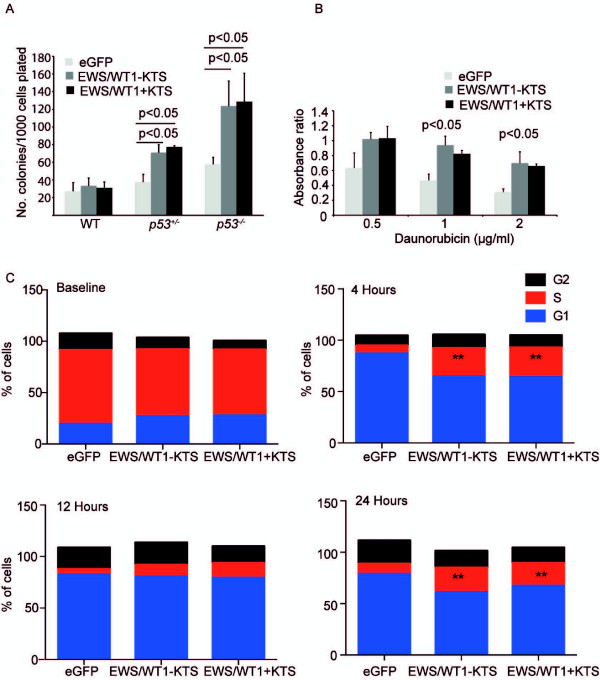
**EWS/WT1-KTS and EWS/WT1 + KTS increase anchorage independent growth and confer resistance to daunorubin induced apoptosis and cell cycle arrest following radiation. (A)** 1x10^4^ p53 wild-type, p53^+/-^ or p53^-/-^ MEFs expressing eGFP, EWS/WT1-KTS or EWS/WT1 + KTS were plated in soft agar and the number of colonies greater than 2 mm counted after 14 days. Values are mean ± SEM of three independently generated and infected pools of MEFs for each genotype tested in three independent experiments. P values of less than 0.05 as determined by Student’s t-tests are shown. **(B)** Viability assay of cells treated with daunorubicin (0.5 μg/ml) for 24 hours. The data show the ratio of WST1 absorbance of treated cells to the same number of untreated cells. Values are mean ± SEM of three independent experiments. P values < 0.05 as determined by Student’s t-tests are shown. **(C)** Wild-type MEFS were irradiated with 10Gy gamma radiation and cell cycle analysis performed at the indicated time points by staining cells with hypotonic propidium iodide solution. Cell-cycle analysis was performed using FACS analysis and the Modfit software package. % of cells in G0/G1, S phase and G2 at baseline, 4, 12 and 24 hours post radiation are shown. Values represent mean of three independent experiments. ** represent p values <0.05 in the comparison of % of cells in S phase and G1 compared to eGFP controls, as determined by Student’s t-tests.

We assayed anchorage independent growth in MEFs over-expressing EWS/WT1 by culturing wild-type, p53^+/-^ or p53^-/-^ MEFs expressing EWS/WT1-KTS, EWS/WT1 + KTS or eGFP in soft agar and counted the number of colonies greater than 2 mm diameter present after two weeks (Figure 3A). As expected, the rate of anchorage independent growth in wild-type MEFs over-expressing eGFP, was very low (0.2% of all cells plated). The deletion of both copies of *p53* resulted in a subtle, EWS/WT1-independent increase in numbers of colonies. Over-expression of EWS/WT1-KTS or EWS/WT1 + KTS, in p53^+/-^ and p53^-/-^ MEFs, significantly increased the number of colonies compared to cells expressing eGFP. These data show that EWS/WT1 co-operates with p53-deletion to also promote anchorage independent growth.

### Cells expressing EWS/WT1 are resistant to daunorubicin-induced cell death and radiation induced cell cycle arrest

We hypothesized that expression of EWS/WT1 may also confer resistance to p53-dependent stimuli, such as apoptosis in response to chemotherapeutic drugs or cell cycle arrest following gamma-irradiation. We used daunorubicin in these experiments, as an example of a well-known cytotoxic chemotherapeutic agent.

Wild-type MEFs expressing eGFP, EWS/WT1-KTS or EWS/WT1 + KTS were treated with daunorubicin and cell viability determined using a tetrazolium salt (WST-1) cell viability assay (Figure 3B). In this assay, an absorbance value of 1 indicates equal numbers of viable cells in treated and control samples. Values less than 1 result from cell loss in treated cells and values greater than 1 indicate more viable cells and proliferation in treated cells. In wild-type MEFs, daunorubicin reduced cell viability in a dose dependent manner (Figure 3B). EWS/WT1 expression significantly reduced the drop in absorbance at each dose compared to eGFP controls, conferring partial protection to daunorubicin. The p53 dependent nature of treatment with daunorubicin was confirmed by treating p53^-/-^ cells, where minimal toxicity was observed following treatment with the same doses of daunorubicin (Additional file 1: Figure S1).

Wild type MEFs were irradiated with 10Gy and cell cycle analysis performed (Figure 3C). Four-hours following irradiation, most eGFP expressing cells had exited the cell cycle and were temporarily arrested in G1. These cells began dividing again by 24 hours post irradiation. In contrast, over-expression of EWS/WT1 + KTS or EWS/WT1–KTS delayed G1 cell cycle arrest to 12-hours and reduced the proportion of cells undergoing G1 arrest. We observed a higher proportion of cells in S-phase in those lines expressing EWS/WT1-KTS or EWS/WT1 + KTS compared to eGFP controls four hours following radiation. EWS/WT1 expressing cells resumed proliferation more rapidly, with an increase in the number of cells in S phase at 24 hours compared to eGFP controls. These observations support the hypothesis that EWS/WT1 over-expression attenuates radiation-induced cycle arrest compared to eGFP controls.

### EWS/WT1 does not block p53 up-regulation in response to daunorubicin

To determine if EWS/WT1 over-expression influenced p53 expression, we probed lysates from wild-type MEFs expressing eGFP, EWS/WT1 + KTS or EWS/WT1–KTS treated with daunorubicin with antibodies to detect p53, p21 and p27 (Additional file 2: Figure S2). p53 expression levels in untreated cells (time 0) was similar in all cells. No difference in p53 up-regulation was observed in cells expressing EWS/WT1 after daunorubicin treatment compared to eGFP controls. These data indicate that while EWS/WT1 expression appeared to attenuate p53 function, it did not directly block p53 expression.

### MDM2 and MDM4 copy number amplification in DSRCT

**Figure 4:**
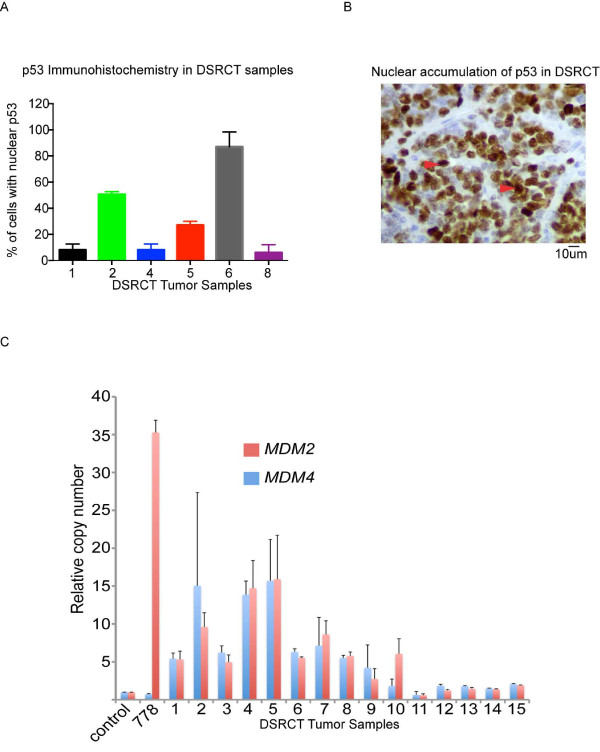
**DSRCT have evidence of nuclear immuno-reactivity of p53 and evidence of *MDM2/MDM4 *copy-number amplification in DSRCT. (A)** % of cells with p53 nuclear immuno-reactivity in five samples of DSRCT stained with anti-p53. The number of cells with nuclear localization of p53 was scored in at least five (5–10) independent fields (20× magnification). Values represent mean ± SEM. **(B)** Representative image of DSRCT sample with anti-p53 immuno-staining. Arrows depict examples of cells with nuclear p53 staining. **(C)***MDM2*/*MDM4* copy number analysis as determined by qPCR in 15 primary DSRCT tumors (#1-15). Data represent the copy number (average and standard deviation) for each sample relative to a normal female blood DNA control (normal control) and the T778 liposarcoma cell line that has *MDM2* amplification. Real-time quantitative PCR was performed using primers for *MDM2*, *MDM4*, or a non-amplified region of Chr17 (as an internal normalisation control for each sample).

Our data suggest that the oncogenic functions of EWS/WT1 are evident when tumor suppressor function of p53 is lost. We screened 15 samples of DSRCT (Additional file 3: Table S1) for the presence of p53 mutations. In this series of tumors, no loss-of-function mutations or deletions in p53 were detected (data not shown). Sections from six of these tumors were available for immunohistochemistry for p53 analysis; three of them were found to have nuclear accumulation of p53 in greater than 20% of tumor cells (Figure 4A and 4B).

Copy-number amplifications of *MDM2* and *MDM4* have been frequently observed in sarcomas and are an important mechanism of p53 inactivation [29]. We used a quantitative PCR assay to measure *MDM2* and *MDM4* copy-number levels. We found copy-number gains and amplification of *MDM2* and/or *MDM4* in at least ten out of fifteen tumors examined (Figure 4C). Ten tumors (66%) had copy number gain of *MDM2* (>5 fold increase) and eight (53%) had a greater than five fold increase in copy number of *MDM4*. Five tumors (33%) had high-level copy number amplification of *MDM2* with greater than ten fold increase and six (40%) had amplification of *MDM4* with greater than ten fold increase. Three of the 15 tumors (20%) had high-level co-amplification of both *MDM2* and *MDM4* (greater than ten fold increase in copy number). These data confirm the presence of copy-number gains or amplifications of *MDM2* and/or *MDM4* in DSRCT.

### Expression profiling of MEFs expressing EWS/WT1-KTS or + KTS identifies canonical Wnt-pathway activation

We performed expression profiling to determine how each EWS/WT1 isoform altered gene-expression in MEFs compared to eGFP controls. Four independently generated pools of wild-type MEFs were infected with doxycyline repressible EWS/WT1 + KTS, EWS/WT1-KTS or eGFP, selected with hygromycin and total RNA harvested and analysed using Illumina MouseWG-6_V2 Expression BeadChips (see Methods). Hierarchical clustering showed that the individual infections within each pool of MEFs, that is, eGFP and EWS/WT1 + KTS or EWS/WT1–KTS were closely related to one another, and that samples clustered according to the embryo from which the MEFs were generated (Additional file 4: Figure S3). Multidimensional scaling and proportion of variance analysis after correction for embryo from which cells were generated indicated that samples next clustered according to the transgene over-expressed (Additional file 4: Figure S3B).

**Figure 5:**
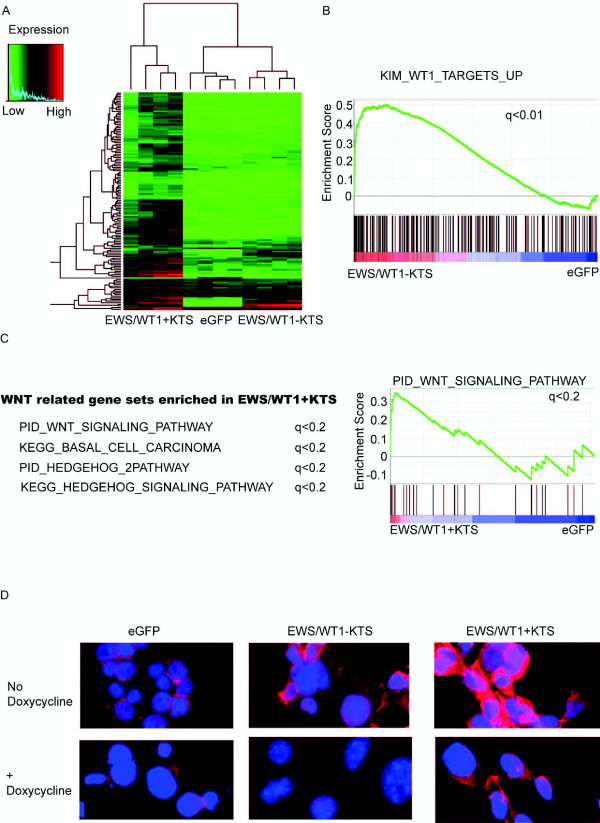
**MEFs expressing EWS/WT1-KTS or EWS/WT1** **+** **KTS have distinct expression profiles and EWS/WT1** **+** **KTS expression is associated with up-regulation of canonical Wnt pathway signaling. (A)** Heat map of differentially expressed probes identified in primary MEFs expressing EWS/WT1 + KTS, eGFP controls and EWS/WT1-KTS. The comparison is of four independently generated pools of MEFs each infected with EWS/WT1 + KTS, EWS/WT1-KTS or GFP. **(B)** GSEA plot depicting enrichment of the KIM_WT1_TARGETS_UP gene set in lines expressing EWS/WT1-KTS compared to eGFP controls. **(C)** Wnt related gene sets enriched in EWS/WT1 + KTS expressing cells (left) and GSEA plot of PID_WNT_Signaling_Pathway geneset (right). **(D)** Immunocytochemistry of 293 T cells infected with doxycycline repressible eGFP, EWS/WT1-KTS or EWS/WT1 + KTS. Cells are shown in the absence of doxycyline (panel on top and also with the presence of doxycycline treatment (thus doxycycline repressed) for 48 hours (panel on bottom). Cells are stained with DAPI for nuclear staining (blue) and β-catenin antibody with secondary anti-goat Alexa Fluor (red). β-catenin translocation from the cellular membrane to the nucleus is a marker of canonical Wnt-pathway activation.

Analysis of differentially expressed genes confirmed that expression of EWS/WT1-KTS or EWS/WT1 + KTS results in distinct gene expression profiles compared to each other, and also compared to eGFP controls (Figure 5A and Additional file 5: Table S2). Eleven genes were differentially expressed between cells expressing eGFP compared to cells expressing either EWS/WT1 isoform, 59 genes were differentially expressed between eGFP cells and EWS/WT1-KTS cells and 132 genes were differentially expressed between eGFP cells and EWS/WT1 + KTS cells (p value < 0.05, q value < 0.1; Additional file 3: Table S1). None of the previously reported targets of EWS/WT1-KTS (PDGFA1, IGFR1, TALLA-1, BAIAP3, ENT4) or EWS/WT1 + KTS (LRRC15 or ENT4) were observed to be differentially expressed in this analysis, using our defined threshold of q value < 0.1.

We performed an unbiased screen of pathways altered by expression of EWS/WT1-KTS or EWS/WT1 + KTS (using the Gene Set Enrichment Algorithm and the C2 (CP) set of signatures with addition of WT1 gene sets [25]) compared to eGFP controls. We found significant alteration of 58 pathways (p value < 0.05, q value <0.25; Additional file 6: Table S3) between cells expressing eGFP and either isoform of EWS/WT1. We observed alteration of 171 pathways in cells expressing EWS/WT1-KTS compared to eGFP controls and 17 pathways in cells expressing EWS/WT1 + KTS (Additional file 6: Table S3).

The KIM_WT1_TARGETS_UP gene set was the 9^th^ most enriched pathway in cells expressing EWS/WT1-KTS compared to eGFP controls (q value <0.01, Additional file 6: Table S3 and Figure 5B) however this pathway was not found to be significantly enriched in cells expressing EWS/WT1 + KTS compared to eGFP controls. This is not surprising as the KIM_WT1_TARGETS_UP genes set was defined largely by genes up-regulated by over-expression of the WT1-KTS isoform [25]. This finding, however, confirms overlap between EWS/WT1 and WT1 target genes.

Four of the 17 pathways enriched in cells expressing EWS/WT1 + KTS involved genes from Wnt and Sonic Hedgehog activation pathways (Figure 5C). There is significant overlap of genes in the Wnt and Sonic Hedgehog genesets, and indeed interactions between the functions of these gene sets [30]. Wnt7b was observed to be in the top 40 genes up-regulated in cells expressing EWS/WT1 + KTS compared to eGFP.

We therefore hypothesized that expression of EWS/WT1 + KTS would result in up-regulation of canonical Wnt-pathway signaling. We used 293 T cells, which have minimal endogenous Wnt pathway activation, to overexpress eGFP, EWS/WT1-KTS or EWS/WT1 + KTS using our doxycyline-repressible lentiviral expression system to examine cellular localization of β-catenin using fluorescence microscopy (Figure 5D). Nuclear β-catenin localization is a marker of activation of canonical Wnt-pathway signaling. We found that in eGFP control cells over-expressing cells, β-catenin was predominantly localized to the cell membrane, with minimal nuclear localization. In the presence of EWS/WT1-KTS expression, there was increased expression of total β-catenin compared to eGFP controls, however this did not appear to be localized to the nucleus. When EWS/WT1 + KTS was expressed, total expression of β-catenin was also increased compared to eGFP controls, however there was also evidence of increased β-catenin localization in the nucleus, consistent with Wnt-pathway activation. This was diminished when cells were treated with doxycycline. This data suggests that enforced EWS/WT1 + KTS expression results in β-catenin nuclear localization and Wnt-pathway activation. EWS/WT1-KTS also appeared to increase total β-catenin expression compared to eGFP controls, however this was not specific to nuclear localization. The mechanism for the observed over-expression for total β-catenin is yet to be determined.

**Figure 6:**
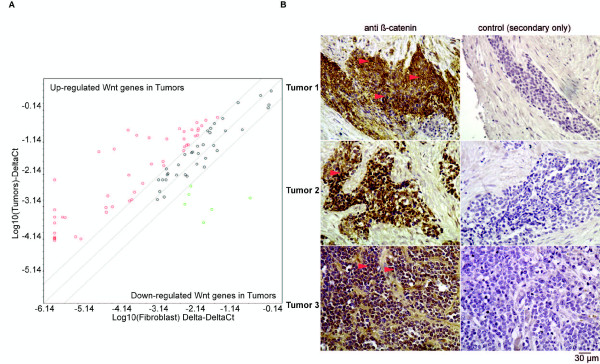
**Canonical Wnt pathway activation in DSRCT. (A)** Scatterplot of fold differences in expression of Wnt-pathway genes in five DSRCT tumors compared to fibroblast control. Lines represent no change in gene expression in DSRCT samples compared to fibroblast controls and these genes are represented by the black symbols. Genes to the left of these lines (red symbols) represent Wnt-pathway genes that are up-regulated in DSRCT samples compared to fibroblast controls while those to the right (green symbols) represent genes that are down-regulated in DSRCT compared to fibroblast controls. **(B)** Representative images of three samples (Tumors 1–3) of DSRCT stained with antibody for β-catenin. Negative antibody controls are shown. Red arrows indicate examples of cells with nuclear reactivity, consistent with canonical Wnt-pathway activation.

We then examined a cohort of five DSRCT tumor samples for which there was sufficient material for evidence of Wnt-activation by quantitative PCR analysis and β-catenin immunhistochemistry. Each of these five tumors expressed both the EWS/WT1-KTS and EWS/WT1 + KTS fusion protein. We found evidence of differential expression of mRNA of 59 Wnt-pathway genes (9 down-regulated) in the five tumors compared to fibroblast controls (Figure 6A and Additional file 7: Table S4). Further, there was evidence of Wnt-activation in these same tumors as determined by nuclear β-catenin immunoreactivity localization (Figure 6B and Additional file 8: Figure S4). Interestingly one of the tumors (Additional file 8: Figure S4C-E) had two distinct populations of cells, one with spindle shaped morphology and the second consistent with the small round cells of DSRCT. Nuclear β-catenin immunoreactivity was restricted to the small round cells, consistent with canonical Wnt-activation in these cells. Taken together these data support the hypothesis that EWS/WT1 results in up-regulation of canonical Wnt-pathway signaling. This is further supported by evidence of Wnt-pathway activation in five DSRCT samples examined.

## Discussion

We have shown for the first time that both isoforms of EWS/WT1 can behave as oncogenes when over-expressed by promoting cell proliferation, anchorage-independent growth, clonogenicity and resistance to apoptotic stimuli in MEFs with loss of p53 function. Analysis of DSRCT demonstrated nuclear-immunoreactivity of p53 and amplification of *MDM2* and *MDM4* in the human tumors, suggesting potential loss of normal p53 tumor suppressor function. In addition, we present what is, to our knowledge, the first evidence of an association between EWS/WT1 + KTS expression and canonical Wnt-pathway signaling.

Neither EWS/WT1-KTS or EWS/WT1 + KTS was sufficient to promote proliferation and survival in wild type MEFs. The oncogenic properties of both, however, were unmasked by loss of p53 function. We thus show the first link between EWS/WT1 function and loss of p53 function. There are several well-described mechanisms by which p53 function is repressed in pediatric tumors including retinoblastoma [31] or sarcoma [32]. These include p53 mutation or copy-number variation of *MDM2* and *MDM4*/*MDMX*[29]. *MDM2* amplification is one of the most frequently observed recurrent gene amplifications in The Cancer Genome Atlas pan-cancer analyses of copy-number variations, and in particular sarcomas [33]. Copy-number amplification of *MDM2* and *MDM4* has also been specifically reported in Ewing’s Sarcoma [29], and co-amplification of both have been observed in a sub-set of sarcomas [34].

The clinical relevance of the finding is reinforced by the studies of the human DSRCT samples. These are necessarily limited in number because of the rarity of the tumor, but nevertheless, a substantial number had *MDM2* and/or *MDM4* copy-number amplification. A limited screen of p53 mutations did not identify well characterized loss-of-function mutants, however a larger and more comprehensive profiling with for example whole exome sequencing would be required to be certain of the true rate of p53 mutations in this tumor sub-type. Thus we cannot exclude the possibility that mutations in other tumor suppressor pathways could also co-operate to drive EWS/WT1 oncogenic function.

DSRCT is a difficult to treat tumor with a dismal prognosis. Loss of p53 function may contribute to this aggressive phenotype. Our data suggested that EWS/WT1 expression contributes to resistance to chemotherapeutic drugs like daunorubicin or gamma irradiation. More successful treatments will require a deeper understanding of the biology of the tumor and further characterization of genomic profiles of these tumors, with for example whole-genome sequencing, may be fruitful.

It is intriguing that we have identified Wnt-pathway activation in our MEF models of EWS/WT1 expression and corroborative evidence for a small number of human samples. The Wnt-pathway is implicated in many human malignancies such as medulloblastoma [35], rhabdomyosarcoma [36], colon cancer [37] and breast cancer [38], through both activation of canonical and non-canonical signaling. This observation can now be pursued in future work to investigate whether the oncogenic properties that we observed in cells expressing EWS/WT1 are dependent on Wnt-activation, and elucidate the mechanism by which EWS/WT1 over-expression activates Wnt-signaling.

Our expression analysis of differentially expressed genes following induction of either EWS/WT1-KTS or EWS/WT1 + KTS did not confirm previously reported targets of the fusion protein. This may reflect differences between our expression system and cell models and those of others. However, an important feature of our project is the inclusion of four independent biological replicates of primary cell lines in an unbiased screen for all differentially expressed mRNAs following over-expression of EWS/WT1. This allowed a pure examination of the effects of EWS/WT1 expression on genome-wide gene expression. An interesting observation in our analysis was of significant enrichment of expression of WT1-KTS associated genes in cells expressing EWS/WT1-KTS, suggesting an overlap in potential target genes of WT1 and EWS/WT1. Further studies focusing on direct transcriptional targets (ChIP-seq) and proteomic analyses will further our understanding of the function of EWS/WT1.

Using the tetracycline repressible lentiviral expression vector we consistently observed higher levels of expression of EWS/WT1 + KTS compared to EWS/WT1-KTS. We therefore cannot conclude whether one splice variant has greater potency in inducing the functional changes observed compared to the other. Nevertheless, in our model system, expression of both EWS/WT1-KTS and EWS/WT1 + KTS was above the threshold required to induce functional effects. It has been shown that the insertion of the KTS amino acids changes the conformation of the DNA binding sites in WT1, and it is likely that the differences in expression profiles observed between cells expressing either EWS/WT1-KTS or EWS/WT1 + KTS is secondary to this.

While we have observed functional changes with over-expression of both isoforms of EWS/WT1 in MEFs, much work remains to be completed in order to understand how these splice variants contribute to tumor formation. It is likely that their oncogenic potential is highly context dependent. In our model system we have examined effect on over-expression in MEFs – in future work it will be important to examine this in other contexts that are more relevant to the human disease, such as over-expression in human mesenchymal stem cells. Finally, the ability of the EWS/WT1 fusion protein to generate tumors *in vivo* remains to be determined in these different contexts.

## Conclusion

In conclusion, we have expanded the knowledge of the function of the EWS/WT1 transgene in DSRCT by showing that both isoforms of the EWS/WT1 can function as an oncogene. We have established novel links between the oncogenic functions of EWS/WT1 and loss of tumor suppressor genes such as p53, and between EWS/WT1 expression and Wnt-pathway activation. These novel insights into the function of EWS/WT1 may provide a way forward for further research of this fusion protein and its role in a rare but lethal malignancy.

## Abbreviations

DSRCT: Desmoplastic small round cell tumor; MEF: Murine embryonic fibroblast.

## Competing interests

The authors declare that they have no competing interest.

## Authors’ contribution

PB, AJ, DMA, EA and PGK conceived of study. Data acquisition was performed by PB, AJ, CR, MS, DP, LR, DNW, DMT, EA and PGE. Analyses were performed by PB, LG, DP, DMA, DNW, DMT, EA and PGE. The manuscript was written by PB, AJ, EA and PGE and all authors were involved in revising it critically for important intellectual content. All authors read and approved the final version.

## Pre-publication history

The pre-publication history for this paper can be accessed here:

http://www.biomedcentral.com/1471-2407/13/585/prepub

## Supplementary Material

Additional file 1: Figure S1 Daunorubicin induced toxicity is p53 dependent in MEFs. Ratio of cell viability of cells treated with daunorubicin (0.5μg/ml) for 24 hours to untreated MEFs of p53^-/-^ MEFs expressing GFP, EWS/WT1-KTS or EWS/WT1 + KTS as measured by colorimetric cell viability assay. Values represent mean ± SEM of three independent experiments performed in independently generated pools of MEFs. Click here for file

Additional file 2: Figure S2 EWS/WT1 does not affect p53 up-regulation following treatment with daunorubicin. Representative western blots of p53, MDM2, p21 and p27 following daunorubicin treatment of wild type MEFs expressing eGFP, EWS/WT1-KTS or EWS/WT1 + KTS. Lysates were generated at time points indicated. Click here for file

Additional file 3: Table S1 Desmoplastic Small Round Cell Tumors used in validation studies. Click here for file

Additional file 4: Figure S3 Hierarchical clustering and multidimensional scaling analysis of gene expression data. **(A)** Hierarchical clustering of four independent pools of MEFs expressing GFP, EWS/WT1-KTS or EWS/WT1 + KTS demonstrate samples are clustered first by embryo from which MEFs were generated. **(B)** Multidimensional scaling demonstrates that after correction for embryo from which MEFs were generated samples cluster according to the transgene which they express. Click here for file

Additional file 5: Table S2 Differentially expressed genes in primary wild type MEFs expressing EWS/WT1-KTS or EWS/WT1 + KTS compared to eGFP controls. Click here for file

Additional file 6: Table S3 Pathways found to be enriched in MEFs expressing EWS/WT1-KTS or EWS/WT1 + KTS compared to eGFP controls on GSEA analysis. Click here for file

Additional file 7: Table S4 Wnt-pathway genes with differential expression in five DSRCT samples compared to human fibroblast control. Click here for file

Additional file 8: Figure S4 DSRCT demonstrate evidence of nuclear β-catenin immunoreactivity consistent with canonical Wnt pathway signaling. **(A)** Sample of DSRCT stained with antibody for β-catenin. Arrows indicate examples of cells with β-catenin nuclear reactivity, consistent with canonical Wnt-pathway activation. **(B)** Control sample of same tumor stained with secondary antibody only. **(C-E)** Representative of a DSRCT that morphologically had two populations of cells, with evidence of Wnt-activation in one. The upper image **(C)** depicts the small round cells characteristic of DSRCT with evidence of β-catenin nuclear reactivity, consistent with canonical Wnt-pathway activation while the lower image **(E)** depicts spindle shaped cells that did not have evidence of activation of Wnt-pathway signaling with cytoplasmic staining of β-catenin. **(D)** Control sample of same tumor stained with secondary antibody only. Click here for file
