# Supplementary figures and images for: The oncogenic properties of EWS/WT1 of desmoplastic small round cell tumors are unmasked by loss of p53 in murine embryonic fibroblasts
Source: BMC Cancer. 2013 Dec 9;13:585. doi: 10.1186/1471-2407-13-585 (PMC4029184; doi:10.1186/1471-2407-13-585)

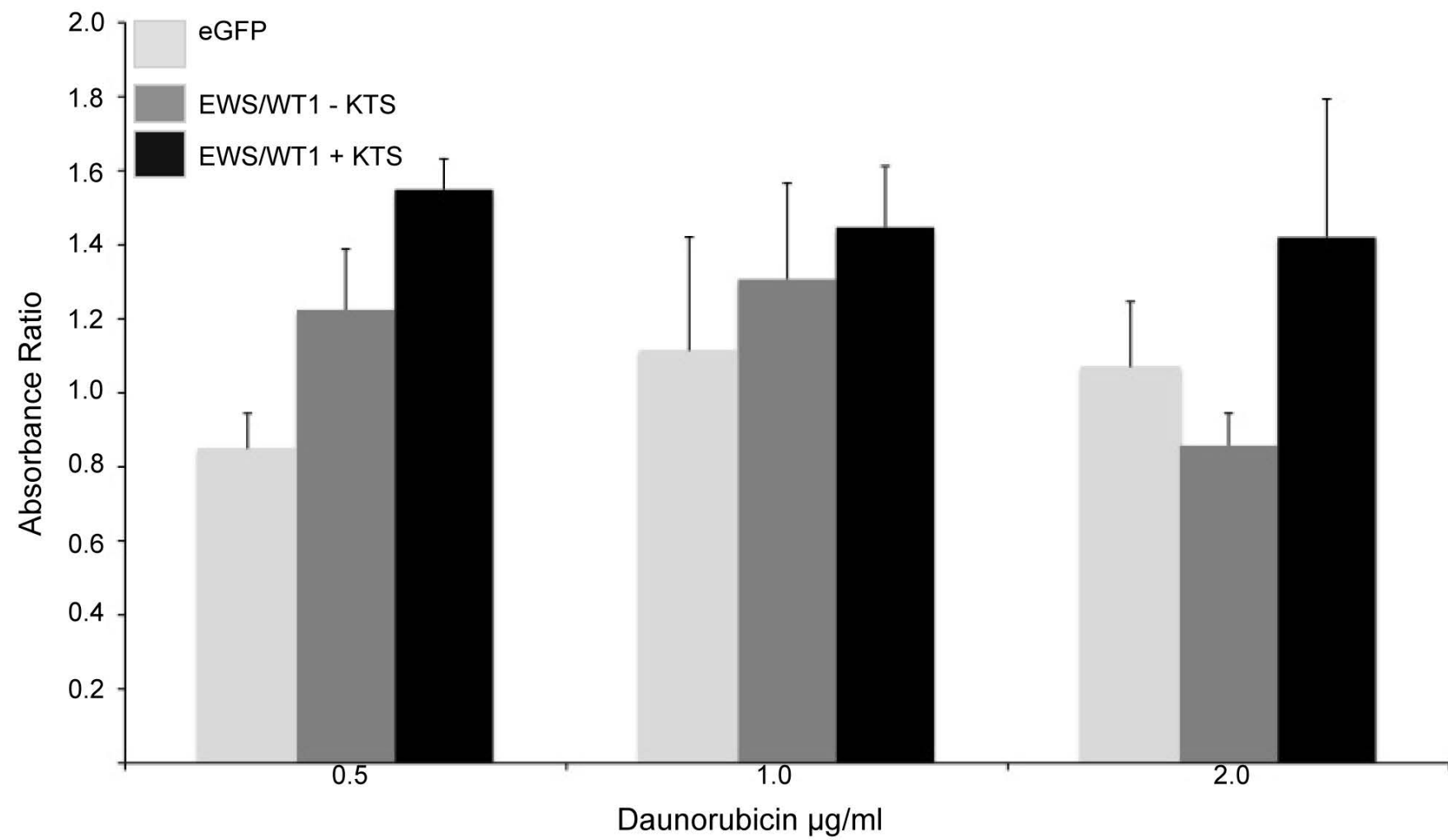

Supplement: Additional file 1: Figure S1 — Daunorubicin induced toxicity is p53 dependent in MEFs. Ratio of cell viability of cells treated with daunorubicin (0.5μg/ml) for 24 hours to untreated MEFs of p53-/- MEFs expressing GFP, EWS/WT1-KTS or EWS/WT1 + KTS as measured by colorimetric cell viability assay. Values represent mean ± SEM of three independent experiments performed in independently generated pools of MEFs. [file 1471-2407-13-585-S1.pdf]

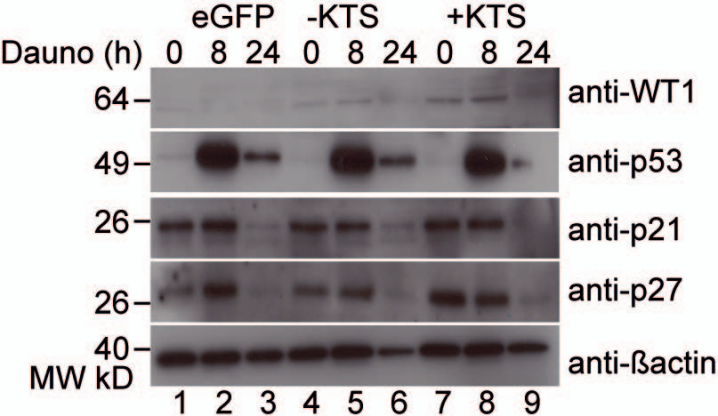

Supplement: Additional file 2: Figure S2 — EWS/WT1 does not affect p53 up-regulation following treatment with daunorubicin. Representative western blots of p53, MDM2, p21 and p27 following daunorubicin treatment of wild type MEFs expressing eGFP, EWS/WT1-KTS or EWS/WT1 + KTS. Lysates were generated at time points indicated. [file 1471-2407-13-585-S2.pdf]

**A**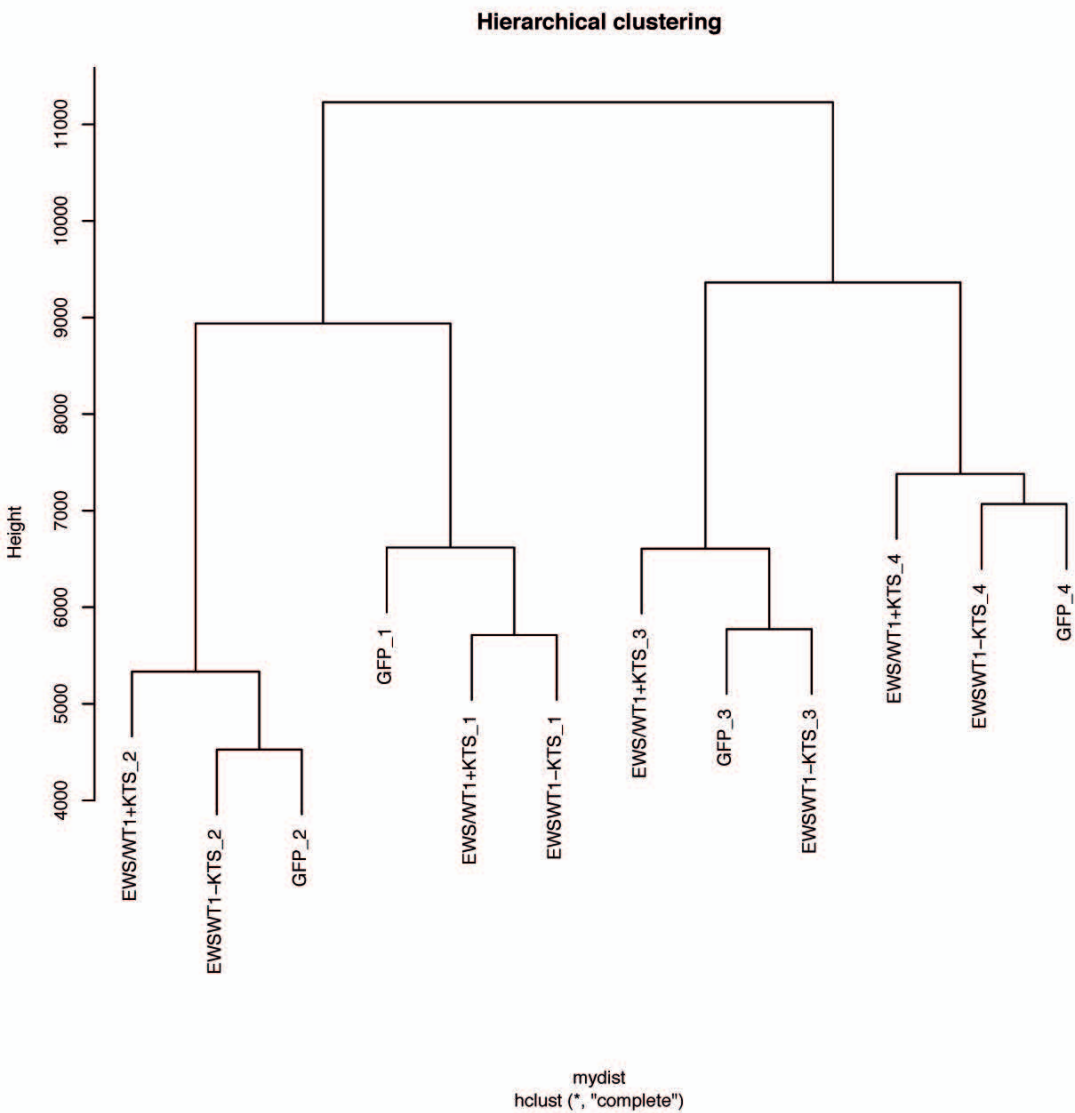**B****Multidimensional Scaling plot of filtered list**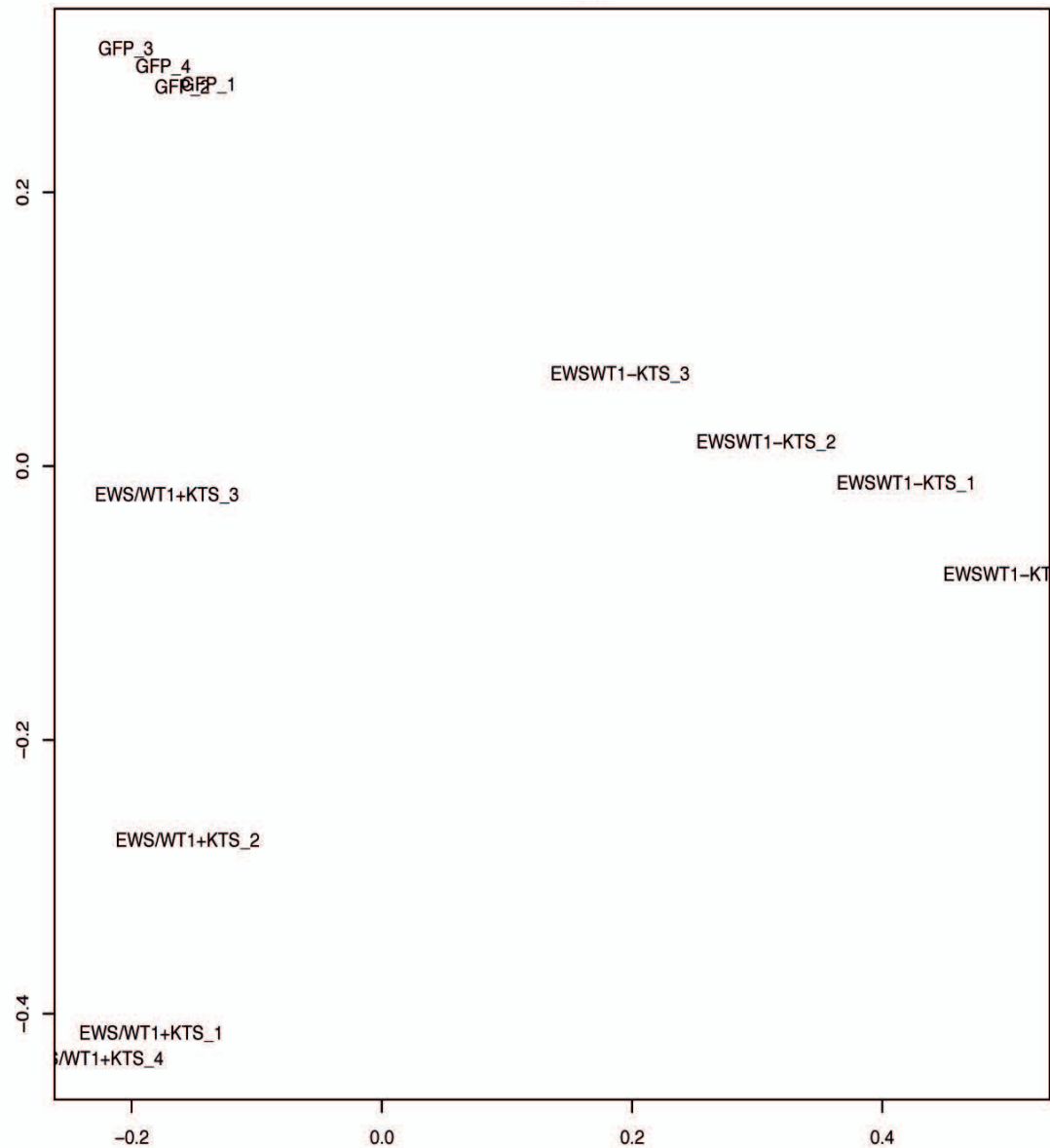

Supplement: Additional file 4: Figure S3 — Hierarchical clustering and multidimensional scaling analysis of gene expression data. (A) Hierarchical clustering of four independent pools of MEFs expressing GFP, EWS/WT1-KTS or EWS/WT1 + KTS demonstrate samples are clustered first by embryo from which MEFs were generated. (B) Multidimensional scaling demonstrates that after correction for embryo from which MEFs were generated samples cluster according to the transgene which they express. [file 1471-2407-13-585-S4.pdf]

**A**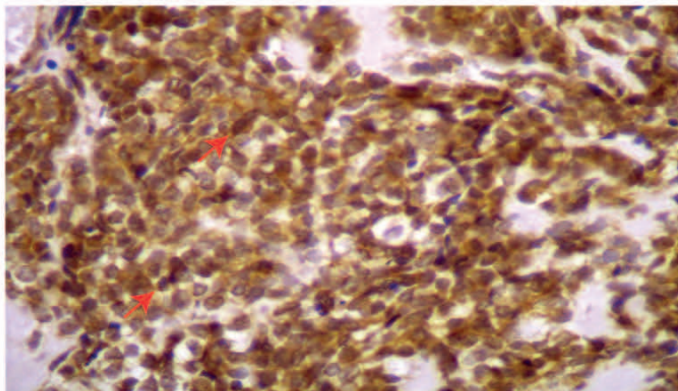**B**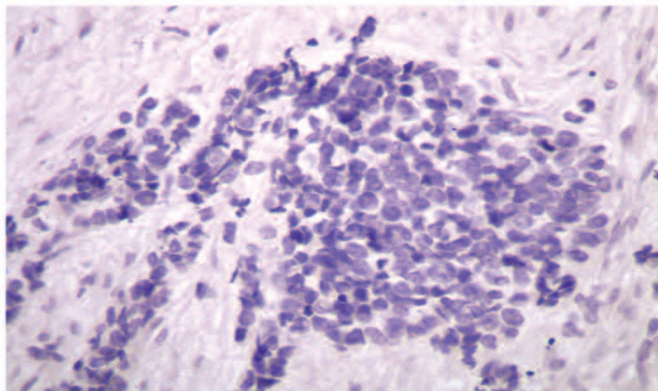

Control (Secondary Only)

**C**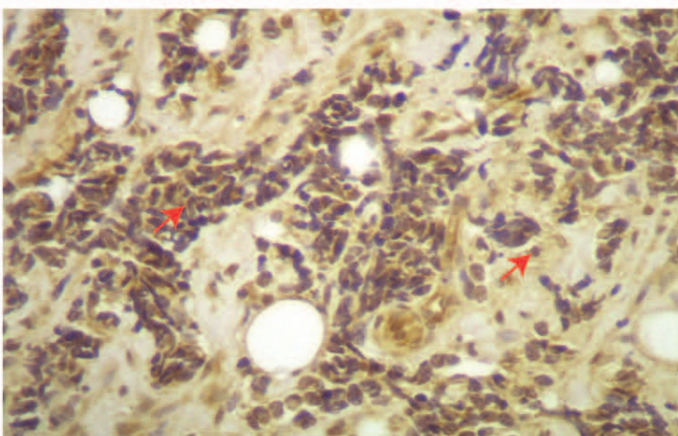**D**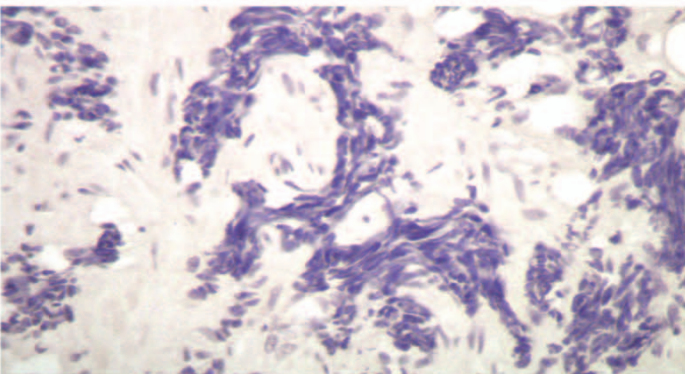

Control (Secondary Only)

**E**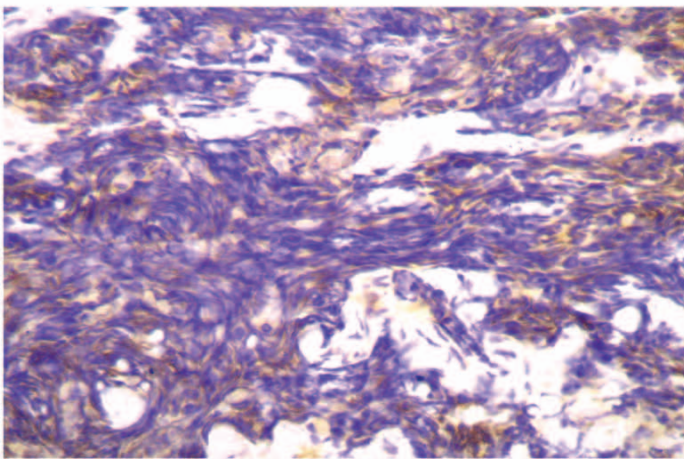

Supplement: Additional file 8: Figure S4 — DSRCT demonstrate evidence of nuclear β-catenin immunoreactivity consistent with canonical Wnt pathway signaling. (A) Sample of DSRCT stained with antibody for β-catenin. Arrows indicate examples of cells with β-catenin nuclear reactivity, consistent with canonical Wnt-pathway activation. (B) Control sample of same tumor stained with secondary antibody only. (C-E) Representative of a DSRCT that morphologically had two populations of cells, with evidence of Wnt-activation in one. The upper image (C) depicts the small round cells characteristic of DSRCT with evidence of β-catenin nuclear reactivity, consistent with canonical Wnt-pathway activation while the lower image (E) depicts spindle shaped cells that did not have evidence of activation of Wnt-pathway signaling with cytoplasmic staining of β-catenin. (D) Control sample of same tumor stained with secondary antibody only. [file 1471-2407-13-585-S8.pdf]
