# Supplementary material for: The oncogenic properties of EWS/WT1 of desmoplastic small round cell tumors are unmasked by loss of p53 in murine embryonic fibroblasts
Source: BMC Cancer. 2013 Dec 9;13:585. doi: 10.1186/1471-2407-13-585 (PMC4029184; doi:10.1186/1471-2407-13-585)
Supplement: Additional file 3: Table S1 — Desmoplastic Small Round Cell Tumors used in validation studies. [file 1471-2407-13-585-S3.docx]

|  | Location | Method of Diagnosis | MDM2/MDM4 qPCR | p53 IHC | Wnt IHC | Comments |  |  |  |
| --- | --- | --- | --- | --- | --- | --- | --- | --- | --- |
| 1 | Intra-abdominal | Morphology | Included | Included |  |  |  |  |  |
| 2 | Intra-abdominal | Morphology | Included | Included |  |  |  |  |  |
| 3 | Intra-abdominal | Morphology | Included |  |  |  |  |  |  |
| 4 | Intra-abdominal | t(11;22) positive | Included | Included |  |  |  |  |  |
| 5 | Intra-abdominal | Morphology | Included | Included |  |  |  |  |  |
| 6 | Intra-abdominal | t(11;22) positive | Included | Included |  |  |  |  |  |
| 7 | Unknown | t(11;22) positive | Included |  |  |  |  |  |  |
| 8 | Intra-abdominal | t(11;22) positive | Included | Included |  |  |  |  |  |
| 9 | Intra-abdominal | Morphology | Included |  |  |  |  |  |  |
| 10 | Intra-abdominal | Morphology | Included |  |  |  |  |  |  |
| 11 | Unknown | t(11;22) positive | Included |  | Included | Both EWS/WT1-KTS and EWS/WT1+KTS expressed | | | |
| 12 | Unknown | t(11;22) positive | Included |  | Included | Both EWS/WT1-KTS and EWS/WT1+KTS expressed | | | |
| 13 | Intra-abdominal | t(11;22) positive | Included |  | Included | Both EWS/WT1-KTS and EWS/WT1+KTS expressed | | | |
| 14 | Intra-abdominal | t(11;22) positive | Included |  | Included | Both EWS/WT1-KTS and EWS/WT1+KTS expressed | | | |
| 15 | Intra-abdominal | t(11;22) positive | Included |  | Included | Both EWS/WT1-KTS and EWS/WT1+KTS expressed | | | |

**Supplemental Table 1: DSRCT samples used in validation**
